# Autophagy induced by avian reovirus enhances viral replication in chickens at the early stage of infection

**DOI:** 10.1186/s12917-019-1926-5

**Published:** 2019-05-24

**Authors:** Xiaosai Niu, Chengcheng Zhang, Yuyang Wang, Mengjiao Guo, Baoyang Ruan, Xuefeng Wang, Tianqi Wu, Xiaorong Zhang, Yantao Wu

**Affiliations:** Jiangsu Co-Innovation Center for Prevention of Animal Infectious Diseases and Zoonoses, College of Veterinary Medicine, Yangzhou University, 12 East Wenhui Road, Yangzhou, 225009 Jiangsu China

**Keywords:** Avian reovirus, Autophagy, IL-1β, Chicken tissues

## Abstract

**Background:**

Avian reovirus (ARV) is an important pathogen that can cause serious disease in poultry. Though several in vitro studies revealed some molecular mechanisms that are responsible for ARV-induced autophagy, it is still largely unknown how ARV manipulates autophagy to promote its own propagation.

**Results:**

In this study, we demonstrated that ARV infection triggered autophagy in chicken tissues, evident from the enhancement of LC3-I/−II conversion and the appearance of abundant autophagosomes. Moreover, viral replication and the expression of IL-1β were coupled with the process of ARV-induced autophagy in the early stage of infection. Furthermore, regulation of autophagy affected the accumulation of LC3-II, the production of ARV and the expression of IL-1β.

**Conclusions:**

Altogether, our data suggest that ARV induces autophagy, which benefits its replication and dissemination in chicken tissues at the early infection stage.

**Electronic supplementary material:**

The online version of this article (10.1186/s12917-019-1926-5) contains supplementary material, which is available to authorized users.

## Background

Avian reovirus (ARV) is an immunosuppressive pathogen [1] that belongs to the *Orthoreovirus* genus in the *Spinareovirinae* subfamily of the *Reoviridae* family [2]. ARV is ubiquitous in poultry flocks, and some strains can lead to severe diseases, causing huge economic losses. The association between viral arthritis and ARV has been conclusively determined. In addition, ARV is also thought to be related to pericarditis, hepatitis, respiratory and enteric disease and malabsorption syndrome [3, 4].

Previous report revealed that ARV-induced apoptosis raises infectious virus yield [5], and several studies focused on the role of apoptosis in the pathogenesis of ARV infection [6, 7]. The tissue injury at the late stage of infection is a likely consequence of ARV-induced apoptosis, but more details about the early stage of ARV infection need to be clarified in order to better control ARV infection. It was reported that ARV-induced autophagy, prior to apoptosis, also promotes its replication [8]. The following researches further illustrated the connection between ARV-induced autophagy and apoptosis, and identified the significance of inhibiting autophagy in the suppression of ARV replication [9–12]. In general, autophagy is an intracellular degradation process that can be triggered to confront viral infections by forming double-membraned autophagosomes targeting cytoplasmic virions or viral components and leading to autolysosomal degradation. On the other hand, viruses have evolved many strategies to manipulate the autophagy pathway, exploiting autophagy machinery for multiplication [13, 14]. Autophagy can be triggered by ARV through specific pathways [11, 15], but how ARV takes advantage of autophagy needs to be further illustrated.

A report emphasized that ARV induced inflammatory response (secretion of IL-1β and IL-6) and delayed apoptosis in the early stage of infection [16]. Another report indicated that activated autophagy can elevate the biosynthesis and secretion of the pro-inflammatory cytokine IL-1β [17]. In addition, our previous research also found that the expression of the receptor of IL-1β (IL-1R) was upregulated in the early stage of ARV infection [18]. All these findings point to a possible connection between ARV-induced autophagy and the inflammatory response, which may be helpful in explaining some symptoms in ARV infection.

As previous studies were all conducted in vitro, verifying whether ARV induces autophagy and whether it is effective to modulate autophagy to control ARV replication in complex in vivo environments are urgent research priorities.

## Results

### ARV infection induced autophagy in chicken tissues

**Fig. 1:**
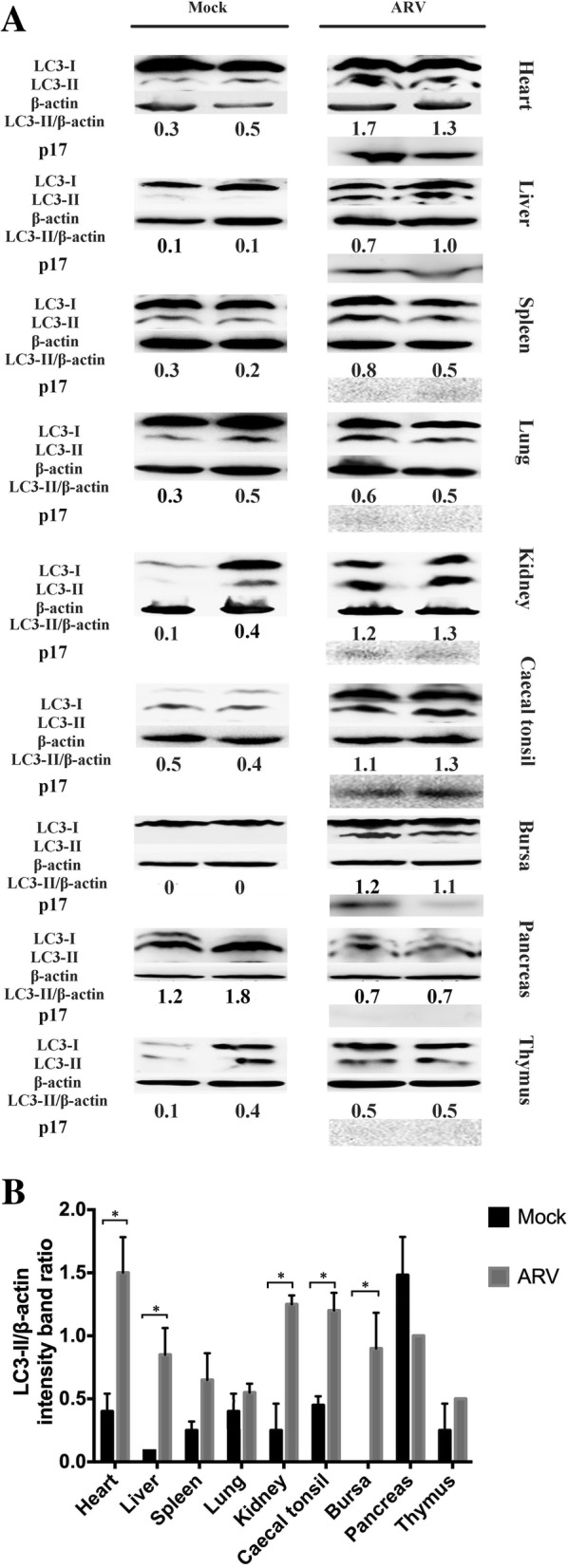
Conversion of LC3-I to LC3-II in different chicken tissues. (**a**) The tissues were collected from Experiment I and were subjected to Western blotting analysis with anti-LC3, anti-β-actin and anti-p17 antibody. Samples of heart, liver, bursa and pancreas were loaded on a ten-lane gel with marker, and other samples were loaded on a parallel gel. (**b**) The relative level of LC3-II in different tissues. β-actin was used as a protein control. Significance was analyzed with multiple t tests (*, *P* < 0.05)

To determine whether autophagy is induced upon ARV infection in vivo, Western blotting analysis was utilized to monitor the change in the abundance of LC3-II protein in the lysates of chicken tissues. After that, ultrastructural analysis of the targeted tissues was performed by Transmission electron microscopy (TEM). The data showed that, compared to uninfected tissues, the conversion from endogenous LC3-I to LC3-II was significantly increased in the heart, liver, kidney, caecal tonsil and bursa of Fabricius from the ARV-infected group (Fig. 1). Though higher amounts of LC3-II were detected in ARV-infected spleen, lung and thymus, the accumulation was not pronounced. However, the basal conversion from LC3-I to LC3-II in mock-infected pancreas was extremely high and even higher than that found in the ARV-infected groups (Fig. 1).

**Fig. 2:**
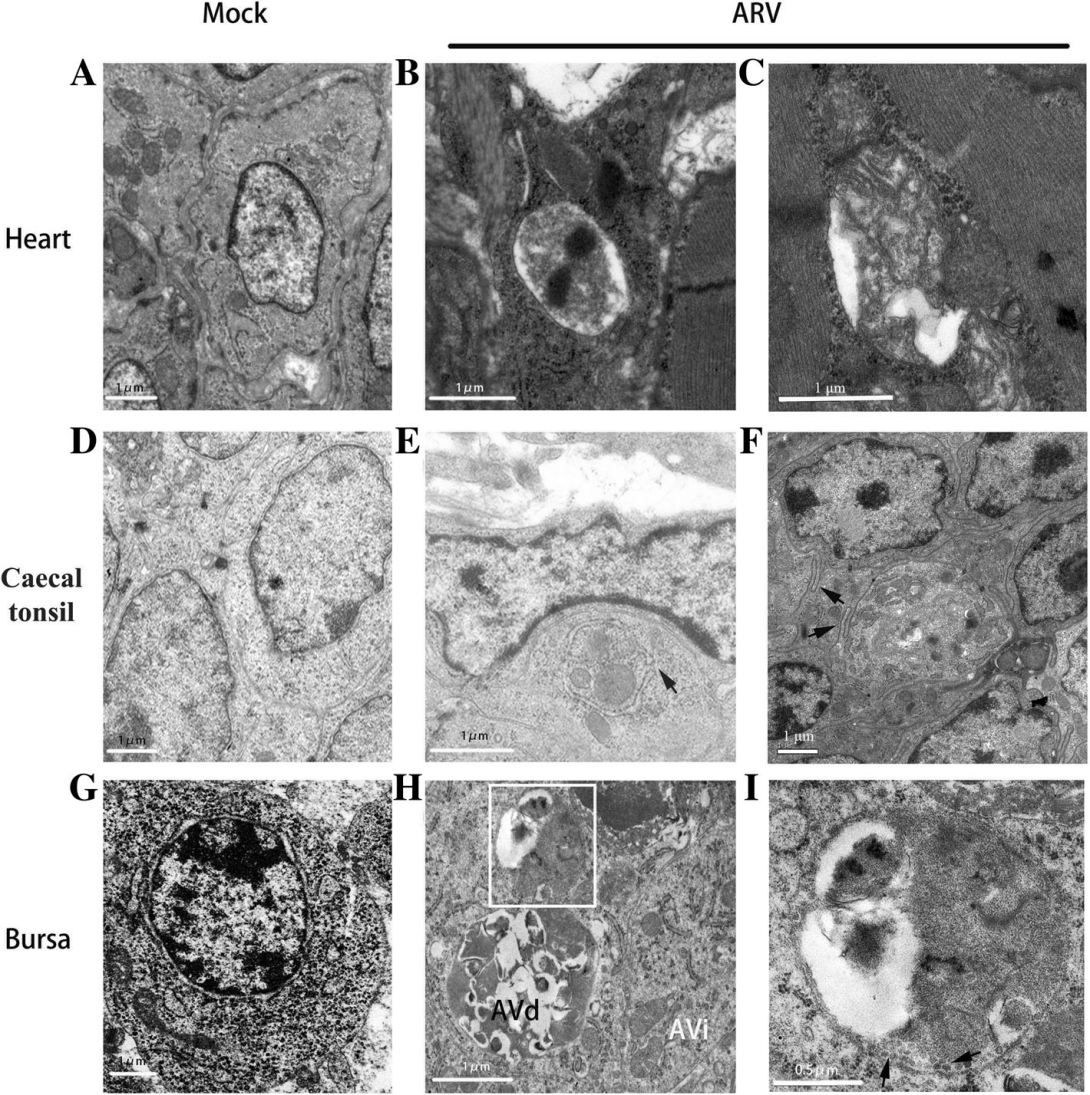
TEM observation of heart, caecal tonsil and bursa. The mock-infected cells (**a**, **d**, **g**) are shown in the first column, and the ultrastructure images of ARV-infected tissues are listed in the second and third columns. Bars, 1 μm (**a** to **h**) and 0.5 μm (**i**). Arrows indicate the growing double membrane

Following collection of data summarized above, TEM was performed on mock-treated and ARV-infected chicken tissues. We examined TEM images of the heart, caecal tonsil and bursa and found that the double-membrane vesicles, in which cytosolic components or organelles were sequestered, were significantly increased in ARV-infected tissues compared with mock-infected tissues (Fig. 2). As depicted in Fig. 2a and c, accumulated autophagosomes were observed in ARV-infected cardiac myocytes and were absent in uninfected cells. Mitochondria with abnormal appearances, such as swelling, disorganization and reduction or vanishing of the cristae, were also observed (Fig. 2b). The gap between myocardial fibers was filled with autophagic structures (Fig. 2c). The amounts of autophagosomes observed in ARV-infected caecal tonsils were also greater than those present in normal cells (Fig. 2d and f). The precursor of the autophagosome, termed the phagophore, in which portions of the cytoplasm and mitochondria were sequestered, was clearly shown in Fig. 2e, with an incompletely closed double-membrane structure (indicated by the arrow). Several autophagic vacuoles at different stages of degradation were discovered in ARV-infected bursal cells, and early initial autophagic vacuole (AVi) and degradative autophagic vacuole (AVd) are shown (Fig. 2h). The AVi can be identified by its contents (morphologically intact cytoplasm, including rough endoplasmic reticulum and mitochondria) and the limiting membrane that is partially visible as two bilayers separated by a narrow electron-lucent cleft. The AVd can be identified by its contents and partially degraded, electron-dense rough ER [19]. The tangentially sectioned inner membrane was also detected (indicated by the arrow in the enlarged Fig. 2i).

### ARV triggered autophagy and promoted the expression of IL-1β along with virus propagation

**Fig. 3:**
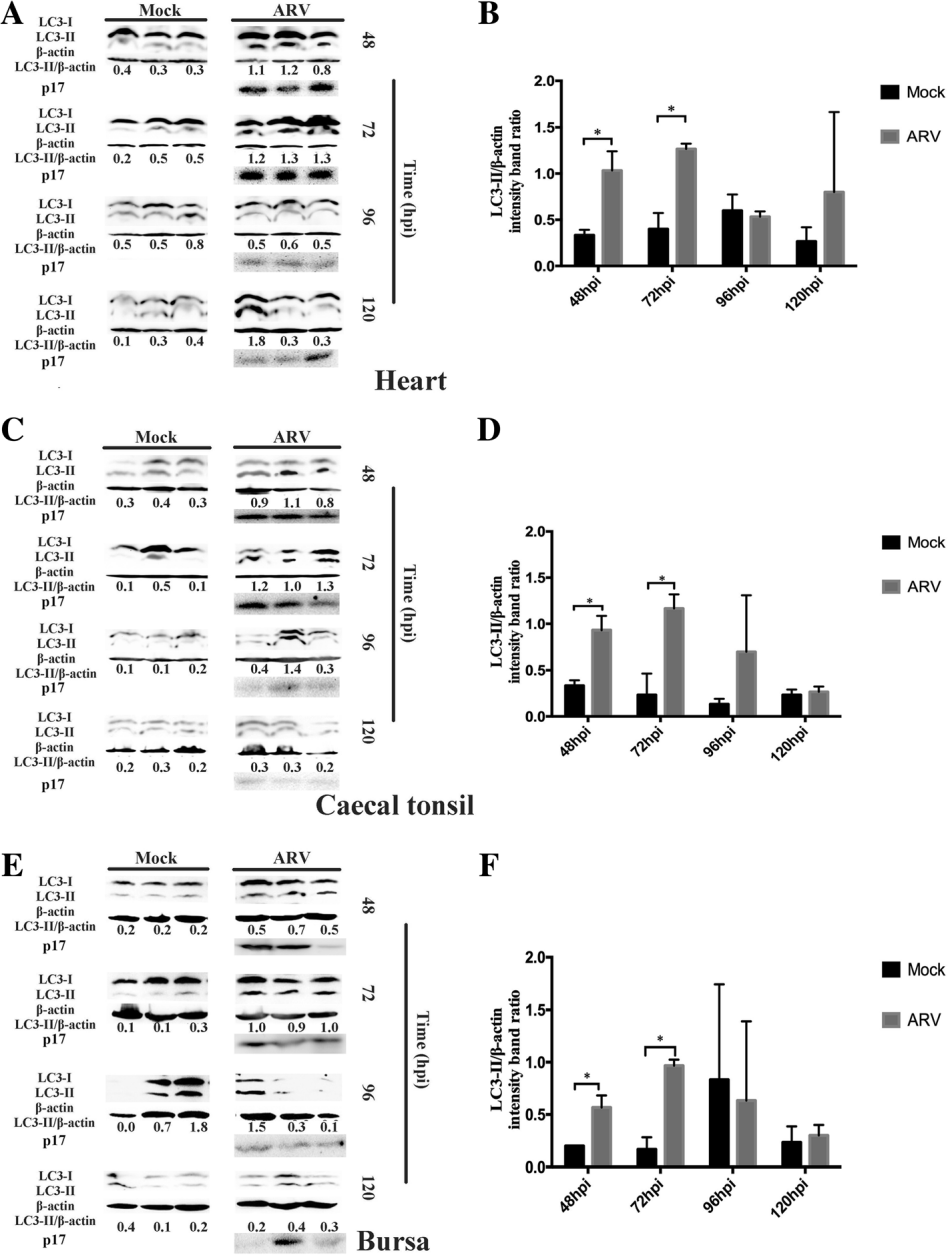
Conversion of LC3-I to LC3-II in heart (**a**, **b**), caecal tonsil (**c**, **d**) and bursa (**e**, **f**) at different time points**.** The tissues were collected from Experiment II at the indicated time points and were subjected to Western blotting analysis. β-actin was used as a protein control and p17 was detected. Significance was analyzed with multiple t tests (*, *P* < 0.05)

To study the development of autophagy in ARV-infected tissues, different time points were set to investigate the accumulation of LC3-II. As shown in Fig. 3, the amounts of LC3-II protein in the heart, caecal tonsil and bursa at different time points were analyzed by Western blotting. In summary, the conversion of LC3-I to LC3-II in ARV-infected tissues reached the highest level at 72 hpi and showed a significant difference compared with uninfected tissues. The increased production of LC3-II could be sustained up to 96 hpi in caecal tonsil (Fig. 3d).

**Fig. 4:**
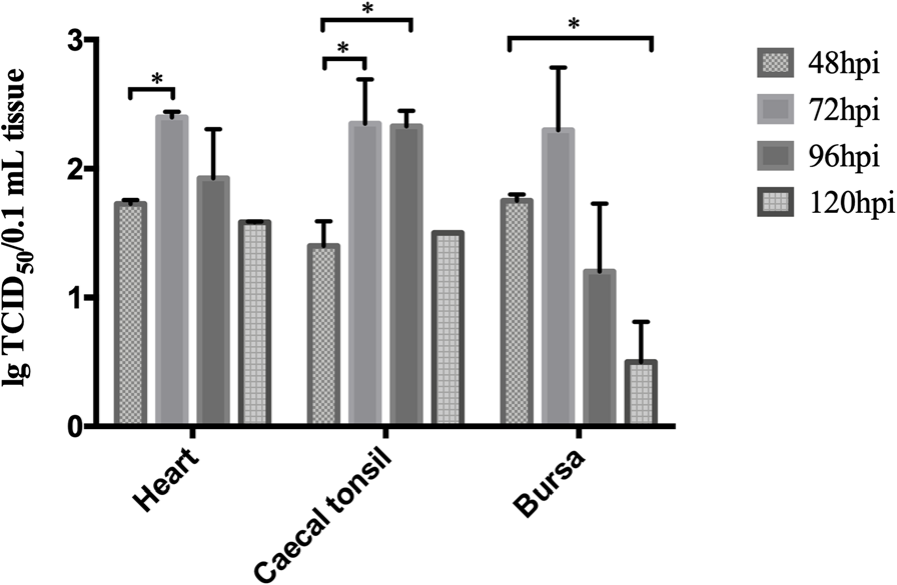
The viral load in ARV-infected heart, caecal tonsil and bursa at different time points. The ARV-infected tissues were collected from Experiment II at the indicated time points, and the virus yields were determined by TCID_50_ in CEF cells. Significance was analyzed with two-way ANOVA (*, P < 0.05)

The viral load in ARV-infected tissues at different time points was determined by TCID_50_ assay. As shown in Fig. 4, ARV replicated well and was most abundant in infected hearts, caecal tonsils and bursae at 72 hpi. At the same time point, autophagy peaked. After 72 hpi, the virus titers showed distinct declining trends. The replication of ARV in the heart dwindled slowly but declined rapidly in bursa. On the other hand, ARV sustained effective replication in the caecal tonsil for a longer period (96 hpi).

**Fig. 5:**
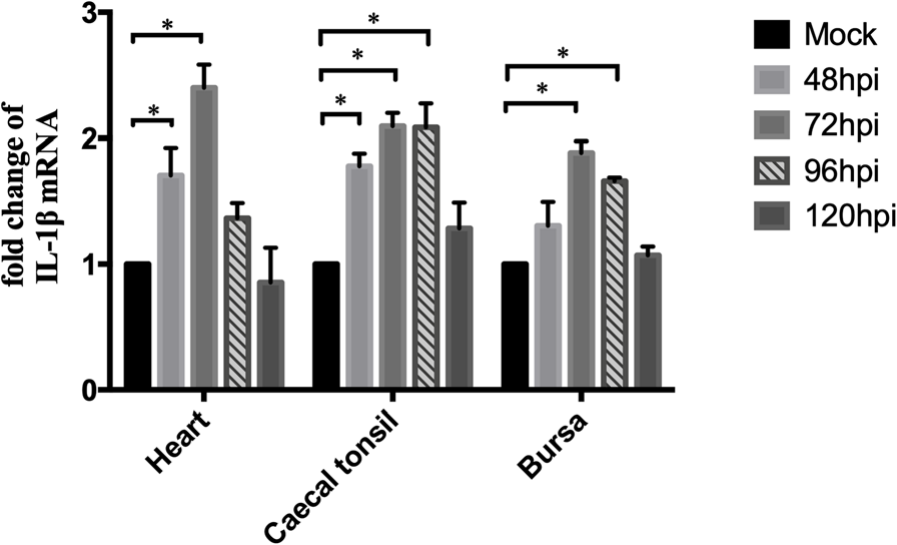
The relative expression levels of IL-1β in heart, caecal tonsil and bursa at different time points. The tissues were collected from experiment II at the indicated time points. The average relative expression levels of IL-1β in mock-infected tissues were designated as 1. The expression levels of IL-1β in ARV-infected tissues are shown as the fold change. Significance was analyzed with two-way ANOVA (*, *P* < 0.05)

To evaluate the pro-inflammatory response in ARV-infected tissues, the expression levels of IL-1β were calculated by RT-qPCR and are shown as fold changes in Fig. 5. The relative expression levels of IL-1β were almost the same across different time points in mock-infected tissues, and the averages were designated as 1. In ARV-infected hearts, the expression of IL-1β reached a peak value at 72 hpi, while the most severe hydropericardium was observed when the chicks were sacrificed at 72 hpi (data not shown). The variation in trends of the relative expression levels of IL-1β in the caecal tonsil and bursa were similar to those in the heart. In the bursa, although the fold value was the lowest, the absolute mRNA quantity of IL-1β was the most abundant (data not shown).

### Modulation of autophagy influenced ARV replication in chicken tissues

The prior data showed a correlation between ARV replication and autophagy, and more experiments were performed to validate the role of autophagy in ARV replication in vivo. Chloroquine (CQ), which can block the formation of autolysosomes and lead to the accumulation of LC3-II at the late stage of autophagy [8], was used as an inhibitor of autophagy. The amounts of LC3-II, the viral load of ARV and the expression of IL-1β in different tissues were calculated as described above.

**Fig. 6:**
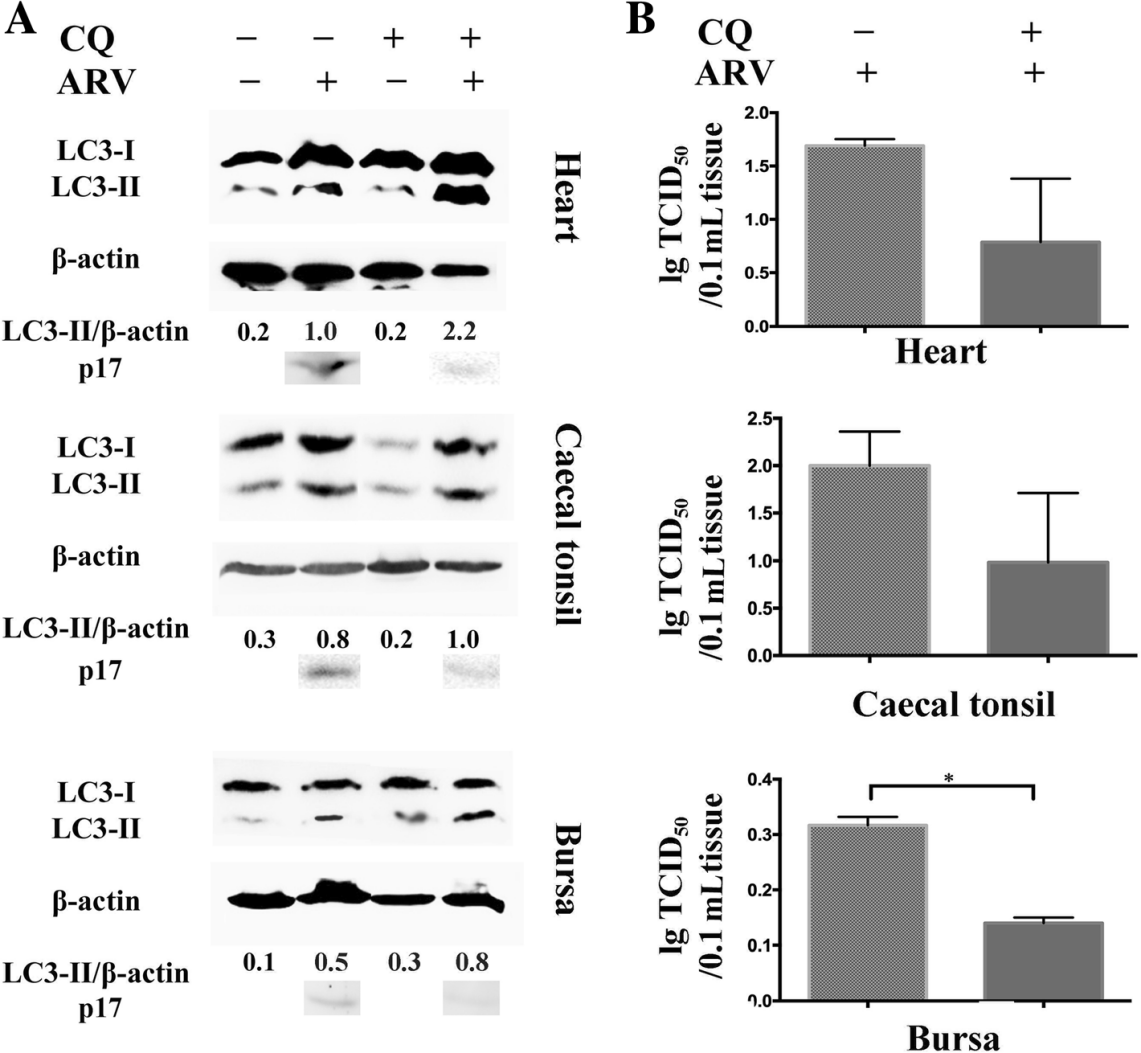
The effect of autophagy on ARV replication in heart, caecal tonsil and bursa. The tissues were collected from Experiment III. The amounts of LC3-II and p17 (**a**) and the viral load (**b**) in heart, caecal tonsil and bursa under different treatments were detected. Significance was analyzed with t tests (*, *P* < 0.05)

**Fig. 7:**
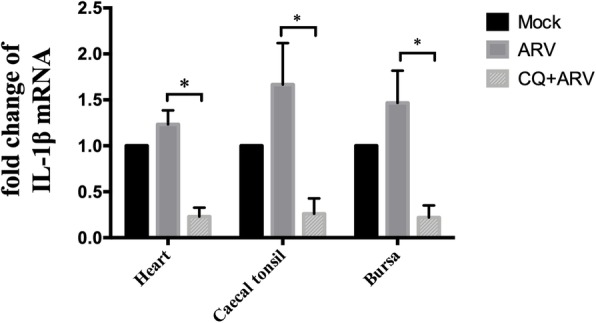
The effect of autophagy on the relative expression levels of IL-1β in heart, caecal tonsil and bursa. The tissues were collected from Experiment III. The relative expression levels of IL-1β in mock-infected tissues were designated as 1. The expression levels of IL-1β in ARV-infected tissues are shown as the fold change. Significance was analyzed with two-way ANOVA (*, *P* < 0.05)

The data (Fig. 6a) showed that ARV-induced the accumulation of LC3-II. Upon treatment with CQ, the degradation of LC3-II was blocked. As shown in Fig. 6b, compared with independent ARV infection, CQ treatment inhibited ARV replication, especially in bursa. Although no significant differences were detected in heart and caecal tonsil between the ARV-infected and CQ-treated groups, CQ treatment reduced the viral load by approximately half compared with independent ARV infection in all checked tissues. Furthermore, CQ treatment remarkably lessened the ARV-induced expression of IL-1β (Fig. 7).

## Discussion

In this study, we examined ARV-induced autophagy in different chicken tissues at the early stage of infection. The LC3 proteins are involved in phagophore formation and are the most widely monitored autophagy-related proteins [19]. The accumulation of LC3-II and the appearance of autophagosomes in ARV-infected heart, caecal tonsil and bursa of Fabricius were particularly notable (Figs. 1 and 2). The intestine-associated lymphoid tissues, such as caecal tonsil and bursa, are thought to be primary viral replication sites after oral inoculation of ARV [6]. Two early studies indicated that ARV could penetrate the intestine by infecting certain epithelial cells, which might be intestinal microfold cells that overlie the lymphoid tissues and interact with the immune system [20, 21]. After prior infection, the replication of ARV and the expression of IL-1β were correlated with the development of autophagy in these immune tissues (Figs. 3 and 5). Autophagy can be stimulated by ARV infection and plays a positive role in ARV replication [8, 14]; then, activated autophagy contributes to the biogenesis and secretion of IL-1β [17]. Consequently, more immune cells are recruited and can be infected [22–24] and carry ARV into the circulatory system.

Pericarditis or myocarditis can be common in ARV infection [3]. The results of this report showed that the conversion of LC3-I to LC3-II was most significant in the heart, and the classical structural features of autophagy (mitochondrial damage) were observed in myocardial tissue (Figs. 1 and 2). A previous study of mammalian reovirus (MRV) identified that MRV only causes cytopathogenic effects in cardiac myocytes but not in cardiac fibroblasts, which is thought to be the determinant of reoviral myocarditis [25]. The severe autophagy combined with the secretion of IL-1β (Fig. 5) may be one of the important inducements of the myocarditis in ARV infection.

Blocking the formation of autolysosomes by CQ led to more accumulation of LC3-II and greatly reduced the viral load in ARV-infected tissues (especially in bursa) (Fig. 6). These results are in accordance with in vitro studies [8, 9]. Except that, CQ treatment significantly decreased the ARV-induced expression of IL-1β in all detected tissues (Fig. 7). The data in this study suggests the relationship between autophagy and ARV infection in vivo, but more detailed mechanisms need to be further studied.

## Conclusions

In conclusion, autophagy is utilized to promote viral replication at the early stage of ARV infection. These results may be helpful for understanding the molecular mechanisms underlying ARV infection and pathology.

## Methods

### Antibodies and reagents

Monoclonal antibody against p17 was prepared by our lab. CQ, antibodies against LC3 or β-actin and horse radish peroxidase-labeled secondary antibodies were purchased from Sigma-Aldrich (Shanghai, China). RIPA lysis buffer, PMSF and SDS-PAGE loading buffer were purchased from Beyotime Biotechnology (Shanghai, China). Chemiluminescent substrate was purchased from Thermo Fisher Scientific (Shanghai, China).

### Cells and virus

Chicken embryonic fibroblast (CEF) cells were prepared from 10-day-old specific-pathogen-free (SPF) chicken embryos and cultured in M199 medium (Gibco, Shanghai, China) supplemented with 3% newborn calf serum (Gibco) and 1% penicillin-streptomycin (Gibco). Cells adhering to plates at 37 °C and 5% CO_2_ were used for cell culture passage. ARV strain GX/2010/1 was propagated in CEF cells, and the titer was determined as the median tissue culture infective dose (TCID_50_) as described previously [26]. The complete genomic sequence of the ARV strain GX/2010/1 was deposited in GenBank (accession numbers KJ476699-KJ476708).

### Animal handling

SPF chicken embryonated eggs were purchased from Boehringer Ingelheim Vital Biotechnology (Beijing, China) and hatched under a controlled temperature (37.8 °C) and humidity (60%). The chickens were housed in cages with free access to food and water during the study. The ARV-infected chickens and the mock-infected chickens were placed in different rooms. The animal experiments were carried out under “Laboratory animals-General requirements for animal experiment” (GB/T 35823–2018, China). The chickens were euthanized by injection of air in intracranial, and the best effort was employed to minimize the pain. A completed ARRIVE guidelines checklist is included in Additional file 1.

### Animal experiments

Experiment I was designed to distinguish whether autophagy would be induced and to measure the levels of autophagy in different tissues after inoculation with ARV. Four 1-day-old chicks were randomly and equally divided into two groups. The ARV-infected group was inoculated orally with 10^7^ TCID_50_ ARV (0.1 mL), and the negative control group was treated with an equal volume of phosphate buffered saline (PBS). At 48 h post inoculation (hpi), the heart, liver, spleen, lung, kidney, caecal tonsil, bursa of Fabricius, pancreas and thymus were collected for further investigation.

Experiment II was designed to estimate the variation tendency of autophagy, the viral load and the inflammatory response with time. Twenty-four 1-day-old chicks were randomly and equally divided into two groups and were treated as previously described in Experiment I. Three chicks from each group were sacrificed every 24 h from 48 hpi. The heart, caecal tonsil and bursa of each chick were collected for Western blotting analysis, viral load determination and reverse transcription-quantitative PCR (RT-qPCR).

Experiment III was designed to verify the roles of autophagy in ARV propagation. Twelve 1-day-old chicks were randomly and equally divided into four groups. Three groups were set as control groups, and the chicks were treated with PBS, CQ (20 mg/kg) or 10^7^ TCID_50_ ARV. Group 4 was set as an autophagy-inhibited group and was treated as described previously [27, 28]. Briefly, the chicks were injected intraperitoneally with CQ (20 mg/kg) 2 h before oral inoculation with 10^7^ TCID_50_ ARV. The injection of CQ (20 mg/kg) was performed every 12 h. At 72 hpi, all chicks were sacrificed and handled as previously described in Experiment II.

### Western blotting

Western blotting was performed as described previously [28] with minor modification in sample preparation. In our research, equal amounts of tissues were collected on ice, and homogenized in ice-cold RIPA lysis buffer supplemented with 1 mM PMSF. The lysates were cleared by centrifugation for 5 min at 12000 rpm at 4 °C, and the supernatants were further denatured by incubation with SDS-PAGE loading buffer at 100 °C for 10 min. Then, equal amounts of protein samples were applied for the Western blotting assay. The immunoreactive bands were incubated with the enhanced chemiluminescent substrate and visualized with a Tanon imager. The LC3 and β-actin were detected on one blot, while p17 was detected on parallel blot. All images were only adjusted with brightness and contrast. Densitometry analysis of the expression of LC3-II and β-actin was performed using ImageJ software (National Institutes of Health, Bethesda, MD, United States). All Western blotting experiments were performed twice.

### Transmission electron microscopy

TEM was used to observe autophagy and could show autophagy in context with its complex cellular environment at subcellular resolution [21]. For ultrastructure analysis, normal and infected tissues, including the heart, caecal tonsil and bursa, were collected from Experiment I and used for TEM observation as described previously [29]. Polymerized thin sections, approximately 60–80 nm, were cut, stained on the grid, examined and photographed with a CM-100 transmission electron microscope (Philips) at 100 kV.

### Viral load determination

After ARV infection, equal amounts of tissues collected from Experiment II and III were mixed and homogenized with 600 μL of PBS. The homogenates were frozen and thawed three times to release the virus. The cellular remains were eliminated by centrifugation at 8000 rpm for 10 min at 4 °C. The supernatants of infected tissues were harvested and serially diluted 10-fold in serum-free medium. These mixtures were used to inoculate CEF cells in 96-well plates to determine TCID_50_ per milliliter of original supernatant.

### RT-qPCR

Total RNA was extracted from the tissues collected from Experiment II using the TRIzon reagent RNA kit (CWbio, Beijing), and reverse transcription was performed for cDNA using the HiScriptII 1st Strand cDNA Synthesis Kit (plus gDNA wiper) (Vazyme, Nanjing) according to the manufacturer’s recommendations. The concentration of total RNA was measured using a spectrophotometer (NanoDrop 2000c, Thermo), and 2 μg of RNA was used for cDNA synthesis in 20 μL volume. The primers for IL-1β and reference gene β-actin used in RT-qPCR were described previously [30, 31] and are listed in Table 1. The optimized PCR mixture consisted of 10 μL Top Green qPCR SuperMix (TransGen, Beijing), 0.25 μM of each primer, 2 μL cDNA and 7 μL H_2_O. The thermal cycling conditions consisted of an initial denaturation step at 94 °C for 20 s, followed by 1 cycle plus 40 cycles of 94 °C for 10 s and 63 °C for 30 s. The melting curve was determined in three steps: 95 °C for 15 s, 60 °C for 1 min, and then heating to 95 °C.

**Table 1:** Primers used in this study

Primer pairs	Sequences	Reference	Standard curves
IL-1β-F	GTGAGGCTCAACATTGCGCTGTA	[28]	y = − 3.45x + 31.16 R = − 1.000, error = 0.005
IL-1β-R	TGTCCAGGCGGTAGAAGATGAAG		
β-actin-F	GAGAAATTGTGCGTGACATCA	[29]	y = − 3.82x + 37.84 R = − 0.998, error = 0.016
β-actin-R	GAGAAATTGTGCGTGACATCA		

To determine the relative mRNA expression level of IL-1β, the standard curves of IL-1β and β-actin were constructed by 1:10 diluted linearized (digested by *Apa*I) standard plasmids based on the *pEASY*-T3 cloning vector (TransGen, Beijing) and are listed in Table 1. The β-actin gene was used as a reference gene for cDNA normalization.

### Statistical analysis

Data were expressed as the means ± standard deviations. Significance was determined with multiple t tests or two-way ANOVA (*, *P* < 0.05). All statistical tests were performed with GraphPad Prism 6.0.

## Additional file

Additional file 1: The ARRIVE Guidelines Checklist. (DOCX 659 kb)

## Data Availability

All data analysed during this study are included in this published article. The raw data generated during the current study are available from the corresponding author on reasonable request.

## Abbreviations

ARV: Avian reovirus
AVd: Degradative autophagic vacuole
AVi: Initial autophagic vacuole
CEF: Chicken embryonic fibroblast
CQ: Chloroquine
hpi: Hours post inoculation
MRV: Mammalian reovirus
PBS: Phosphate buffered saline
RT-qPCR: Reverse transcription-quantitative PCR
SPF: Specific-pathogen-free
TCID_50_: The median tissue culture infective dose
TEM: Transmission electron microscopy
