# Supplementary material for: Autophagy induced by avian reovirus enhances viral replication in chickens at the early stage of infection
Source: BMC Vet Res. 2019 May 24;15:173. doi: 10.1186/s12917-019-1926-5 (PMC6534907; doi:10.1186/s12917-019-1926-5)

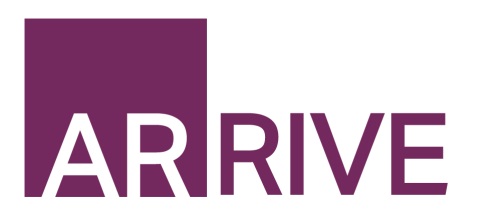


The ARRIVE Guidelines Checklist

Animal Research: Reporting In Vivo Experiments

Carol Kilkenny^1^, William J Browne^2^, Innes C Cuthill^3^, Michael Emerson^4^ and Douglas G Altman^5^

*^1^The National Centre for the Replacement, Refinement and Reduction of Animals in Research, London, UK, ^2^School of Veterinary Science, University of Bristol, Bristol, UK, ^3^School of Biological Sciences, University of Bristol, Bristol, UK, ^4^National Heart and Lung Institute, Imperial College London, UK, ^5^Centre for Statistics in Medicine, University of Oxford, Oxford, UK.*

|  | | ITEM | RECOMMENDATION | Section/ Paragraph |
| --- | --- | --- | --- | --- |
| 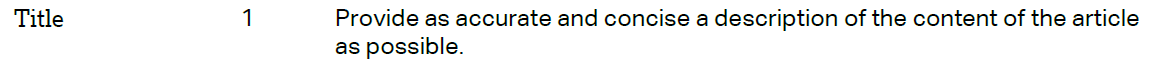 | | | Title |  |
| 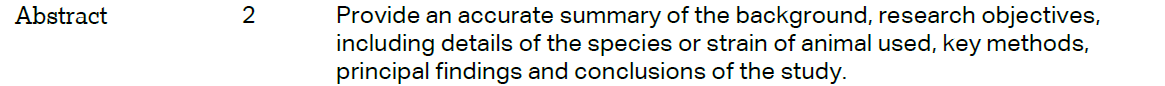 | | | Abstract |  |
| INTRODUCTION | | |  |  |
| 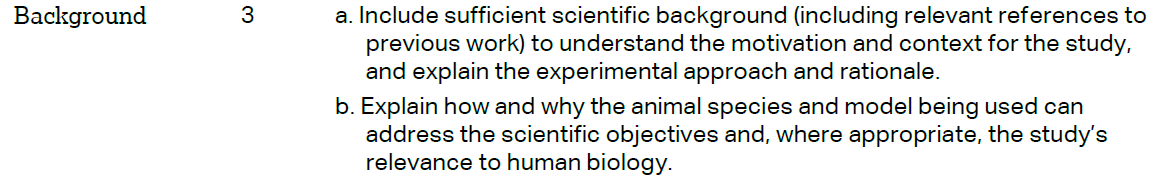 | | | Paragraphs 1-3  Paragraphs 2-3 |  |
| 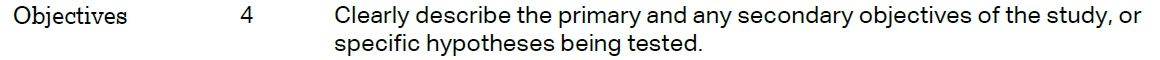 | | | Paragraph 4 |  |
| METHODS | | |  |  |
| 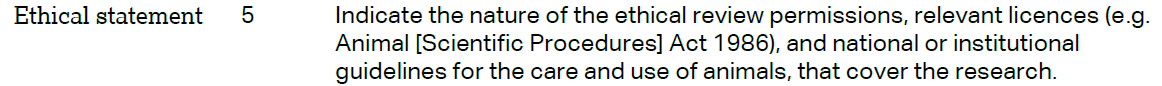 | | | Paragraph 3 |  |
| 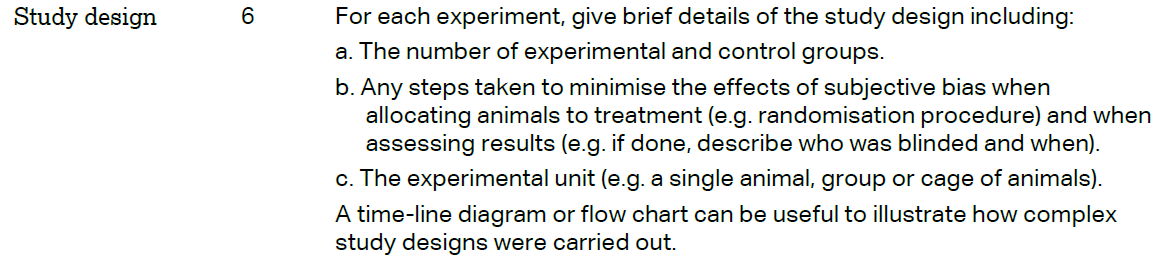 | | | Paragraphs 4-6  N/A |  |
| 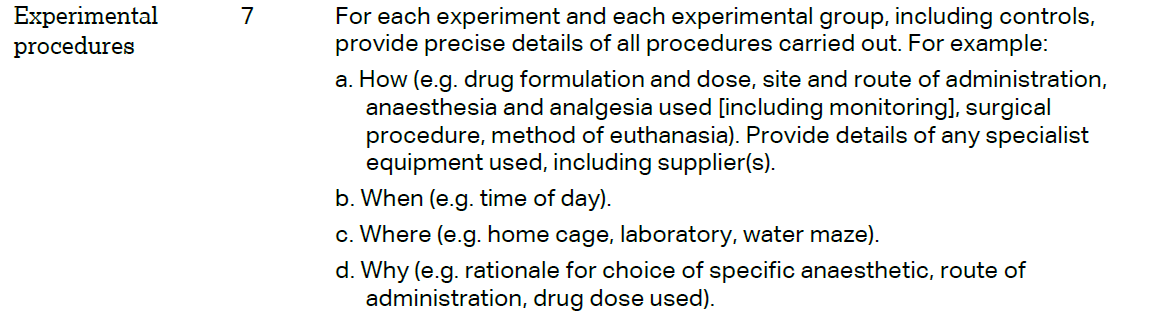 | | | Paragraphs 4-6 |  |
| 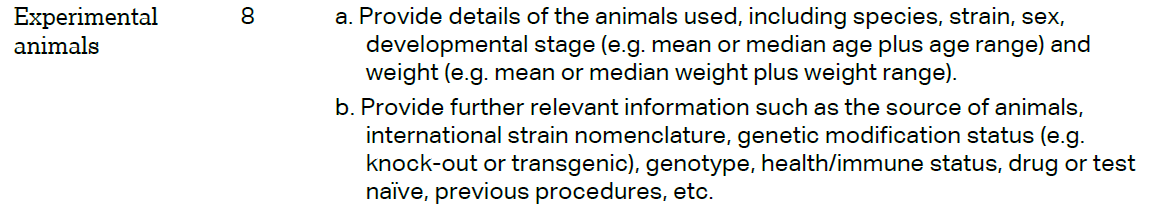 | | | Paragraph 3 |  |

The ARRIVE guidelines. Originally published in *PLoS Biology*, June 2010^1^

| 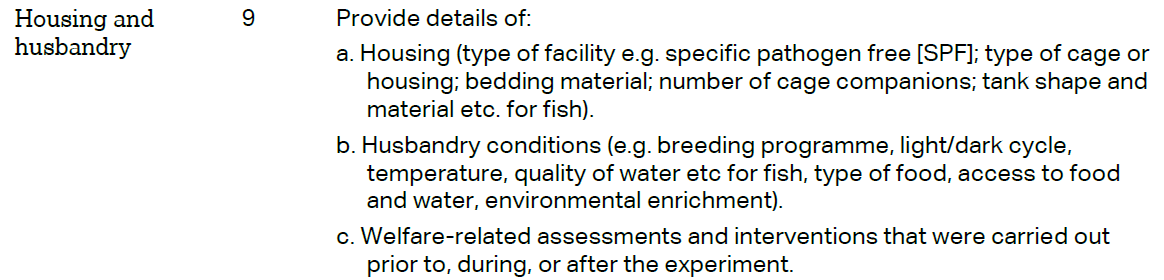 | Paragraph 3 |  |  |
| --- | --- | --- | --- |
| 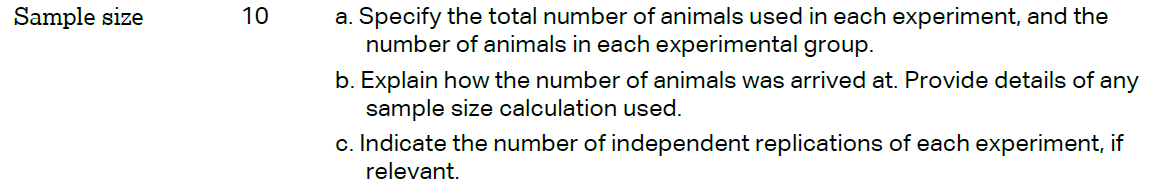 | Paragraphs 4-6 |  |  |
| 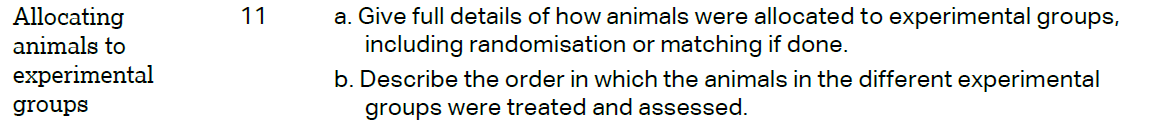 | Paragraphs 4-6 |  |  |
| 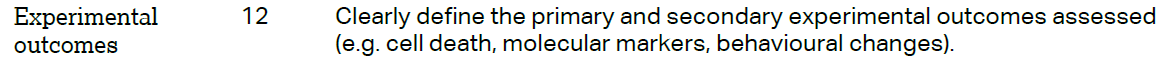 | Paragraphs 7-11 |  |  |
| 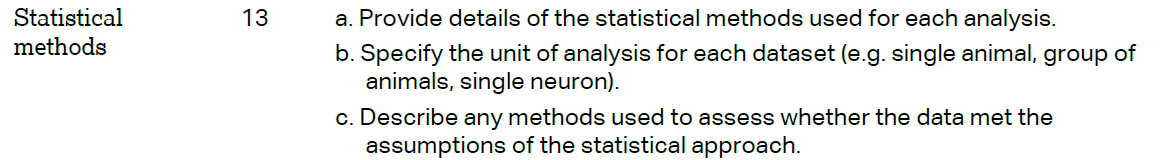 | Paragraph 12 |  |  |
| RESULTS |  |  |  |
| 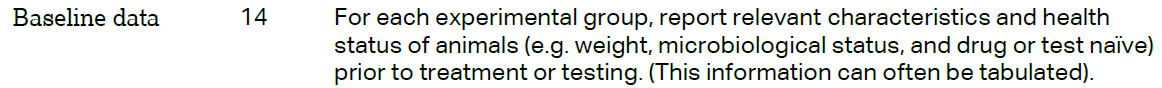 | N/A |  |  |
| 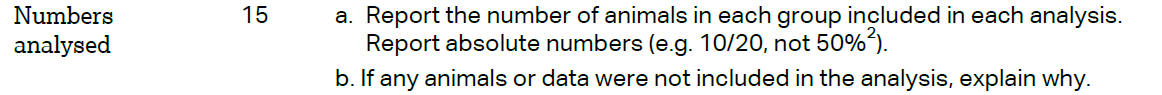 | Methods  Paragraphs 4-6 |  |  |
| 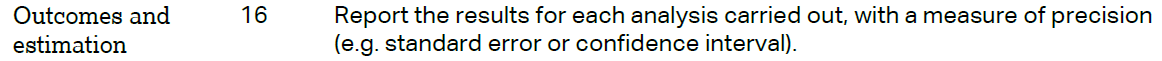 | Figures 1-7 |  |  |
| 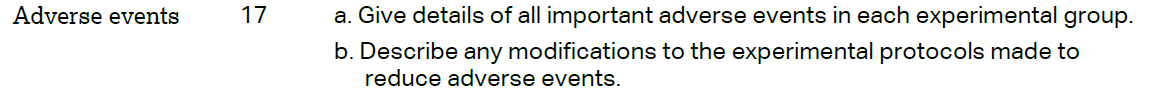 | N/A |  |  |
| DISCUSSION |  |  |  |
| 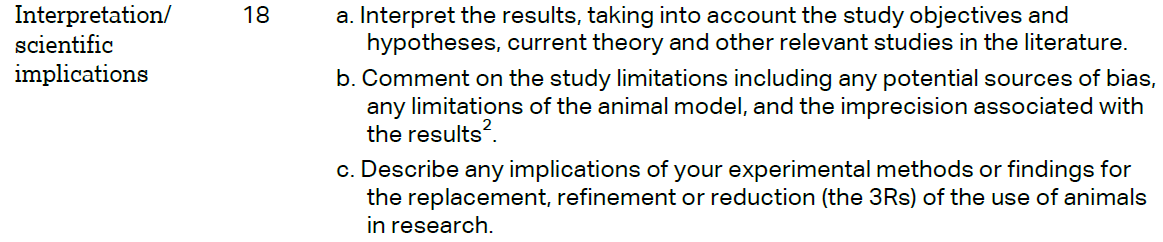 | Paragraphs 1-3  N/A  N/A |  |  |
| 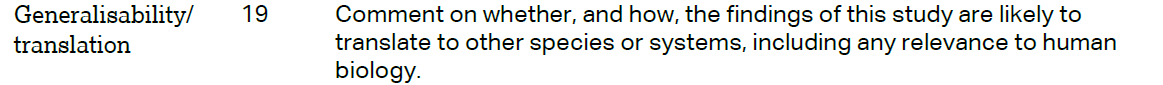 | N/A |  |  |
| 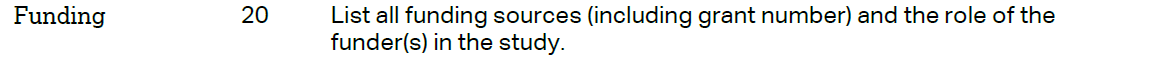 | | Declarations  Funding | |


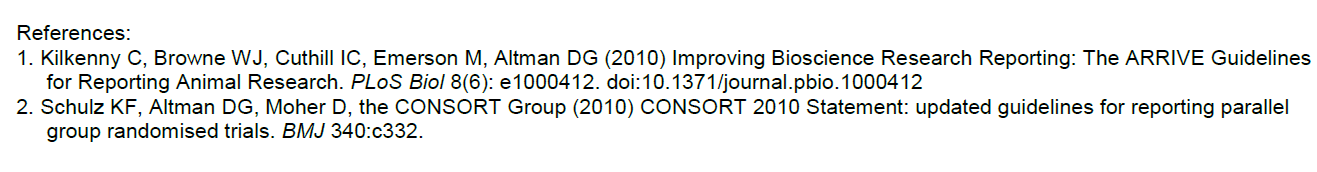

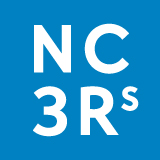

Supplement: Supplementary file 1 — The ARRIVE Guidelines Checklist. (DOCX 659 kb) [file 12917_2019_1926_MOESM1_ESM.docx]
